# Severe Hypercalcemia and Confusion in a Middle‐Aged Male: The Hidden Diagnosis of Parathyroid Carcinoma

**DOI:** 10.1155/crie/5998817

**Published:** 2026-01-20

**Authors:** Helena Fahmi, Mahmoud Karaki, Fatima Yasmin, Layal Akl, Paola Atallah

**Affiliations:** ^1^ Department of Endocrinology and Metabolism, University of Balamand, Beirut, Lebanon, balamand.edu.lb; ^2^ Department of Endocrinology and Metabolism, Saint George Hospital University Medical Center, Beirut, Lebanon, stgeorgehospital.org

## Abstract

Parathyroid cancer (PC) is one of the rarest causes of primary hyperparathyroidism (PHPT), typically exhibiting an indolent course but presenting with more severe symptoms compared to its benign counterparts. The diagnosis is most often made postoperatively through histopathological examination; however, certain clinical and biochemical features may raise suspicion preoperatively. These include markedly elevated serum calcium and parathyroid hormone (PTH) levels, a large parathyroid lesion with suspicious ultrasonographic features, and evidence of renal or skeletal complications. Although the exact etiology remains unclear, somatic mutations in the *CDC73* gene have been identified in patients with PC. Complete surgical resection via en bloc excision remains the first‐line and most effective therapeutic approach to maximize the chance of cure, although recurrence is common during follow‐up. Other treatment modalities, including radiotherapy, chemotherapy, and immunotherapy, have limited evidence supporting their efficacy. Here, we report the case of a 53‐year‐old male who presented with lower limb weakness, confusion, and significant weight loss over the preceding month. His past medical history included prediabetes and dyslipidemia. Initially evaluated by a neurologist for depressive symptoms, he subsequently developed rapidly progressive neurocognitive decline, impaired mobility, and continued unexplained weight loss. Upon hospital admission, he was somnolent and confused, though hemodynamically stable. Laboratory investigations revealed severe hypercalcemia at 18 mg/dL (reference range: 8.5–10.5), acute kidney injury with a creatinine of 4.2 mg/dL (0.7–1.3), and a markedly elevated PTH level of 1095 pg/mL (10–65). Initial management included aggressive intravenous hydration and administration of denosumab to control the symptomatic hypercalcemia, which resulted in improved calcium levels and renal function. Further evaluation with imaging, including parathyroid ultrasound and Technetium‐99m (Tc‐99m) sestamibi scintigraphy, was consistent with a right parathyroid adenoma. The patient subsequently underwent parathyroidectomy, and histological analysis confirmed the diagnosis of parathyroid carcinoma.

## 1. Introduction

Parathyroid cancer (PC) is a rare malignancy of the endocrine system, accounting for less than 1% of all cases of primary hyperparathyroidism (PHPT) [1]. Its incidence is rising, likely due to increased detection through routine serum calcium screening [2]. As with other causes of PHPT, the hypercalcemia observed in PC results from excessive and autonomous secretion of parathyroid hormone (PTH). However, patients with PC typically present with a more symptomatic course, often involving end‐organ damage due to significantly elevated calcium and PTH levels. While most cases of PC are sporadic, it may also occur as part of hereditary syndromes such as hyperparathyroidism‐jaw tumor (HPT‐JT) syndrome [3], multiple endocrine neoplasia type 1 (MEN1) [4], and multiple endocrine neoplasia type 2A (MEN 2A) [5]. Preoperative diagnosis of PC remains difficult, with most cases identified postoperatively based on histopathological findings, including fibrous trabeculae, mitotic activity, capsular invasion, and vascular invasion [6]. Surgical management via en bloc resection, including ipsilateral hemithyroidectomy and central lymphadenectomy, remains the cornerstone of treatment to minimize the risk of recurrence [7].

## 2. Case Presentation

We present the case of a 53‐year‐old male who was admitted to the hospital in May 2022 with a 1‐month history of lower limb weakness, confusion, and unintentional weight loss. His medical history was significant for prediabetes managed with metformin 1000 mg daily and dyslipidemia treated with atorvastatin 10 mg daily. Approximately 1 year prior, he had been evaluated by a neurologist for depressive symptoms. Over the months leading up to admission, he developed rapidly progressive neurocognitive decline, difficulty ambulating, and a 17 kg weight loss despite a preserved appetite. Due to the worsening of his symptoms, the neurologist recommended hospital admission for further evaluation.

On presentation, the patient was somnolent and confused but hemodynamically stable, with a blood pressure of 130/70 mmHg, a heart rate of 92 beats per minute, and an afebrile status. Capillary blood glucose was 89 mg/dL.

Initial laboratory workup revealed the following significant findings:

**Table:** 

Test	Result	Reference range
Hgb	12.5 g/dL	(13–17) g/dL
MCV	86 fL	(80–100) fL
Creatinine	4.2 mg/dL	(0.7–1.3) mg/dL
BUN	82 mg/dL	(6–24) mg/dL
Calcium	18 mg/dL	(8.5–10.5) mg/dL
Phosphorus	2.9 mg/dL	(2.8–4.5) mg/dL
Albumin	3.7 g/dL	(3.4–5.4) g/dL
TSH	0.5 IU/mL	(0.27–4.9) IU /mL
Vitamin B12	885 pg/mL	(160–950) pg/mL
HbA1c	5.3%	(4–5.7) %

He was started on aggressive intravenous hydration for hypercalcemia, and the endocrinology team was consulted for further evaluation.

A thorough review of his medical and family history revealed a previously uninvestigated personal and familial history of recurrent nephrolithiasis. Retrospective laboratory data showed a calcium level of 10.7 mg/dL (reference range: 8.5–10.5) in 2019. Additionally, a thyroid ultrasound conducted in January 2022 for neck discomfort, approximately 4 months before presentation, had shown no evidence of thyroid or parathyroid pathology.

During admission, a CT scan of the chest, abdomen, and pelvis was performed to exclude renal obstruction and identify any secondary causes of hypercalcemia; the scan was unremarkable. Laboratory investigations revealed a markedly elevated PTH level of 1095 pg/mL (reference range: 10–65), serum calcium of 18 mg/dL, vitamin D level of 55 ng/mL, and alkaline phosphatase of 95 IU/L. A repeat calcium level following initial IV hydration was 15 mg/dL. The patient’s mental status began to improve with supportive management, and given his clinical improvement and clear biochemical diagnosis, no further neurological investigations were pursued. A 24‐h urinary calcium collection demonstrated hypercalciuria at 513.4 mg/24h (normal range: 100–300 mg/24h). On day 4 of hospitalization, serum calcium remained elevated at 12.9 mg/dL, prompting administration of subcutaneous denosumab 30 mg. By day 7, renal function had improved, with creatinine reduced to 3.0 mg/dL and serum calcium normalized to 10.0 mg/dL.

**Figure 1:**
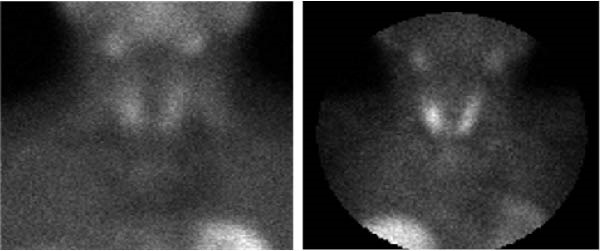
Sestamibi scan demonstrates asymmetric radiotracer uptake in the right cervical region.

Given the previously normal ultrasound findings, Technetium‐99m (Tc‐99m) sestamibi scintigraphy was selected as the initial imaging modality during this admission. The scan demonstrated asymmetric radiotracer uptake in the right cervical region, suggestive of a right parathyroid adenoma (Figure 1).

A working diagnosis of PHPT secondary to a parathyroid adenoma was established. The patient was discharged following stabilization of calcium levels and clinical improvement, with plans for repeat laboratory testing and parathyroid ultrasound as an outpatient. A 4D CT scan was also requested as part of the diagnostic workup, but the patient opted not to proceed with the examination.

**Figure 2:**
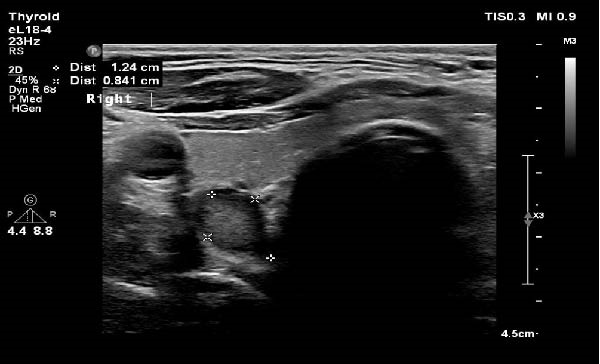
Parathyroid ultrasound demonstrates a well‐circumscribed, 1.2 cm lesion in the right inferior parathyroid region, consistent with a parathyroid adenoma.

During the outpatient follow‐up in June 2022, a parathyroid ultrasound revealed a 1.2 cm right inferior parathyroid adenoma without any suspicious features or lymphadenopathy reported (Figure 2), and the patient was scheduled for a parathyroidectomy.

The surgery was performed on 28/06/2022. Intraoperatively, an enlarged right inferior parathyroid gland was identified, and no other abnormalities were reported in the surgical report. Local excision of the enlarged parathyroid gland was done, and a frozen section was sent, revealing a hypercellular parathyroid, compatible with adenoma. Intraoperative PTH levels dropped from 1086 pg/mL to 68 pg/mL within 20 min, and no further resection was performed. The postoperative course was unremarkable, and the patient was discharged home.

Final histopathological analysis of the excised 1.9 × 1.4 × 0.7 cm parathyroid gland revealed features consistent with parathyroid carcinoma, including lymphovascular invasion and low cytonuclear grade. The report notes that the gland is surrounded by an intact, dense fibrotic capsule, containing some entrapped chief cells that are arranged in nests and aggregates of variable size and shape. In one block (1C), there is a capillary with a clearly visible endothelial cell layer, containing rare blood cells and fibrin partially occupied by a cohesive aggregate of chief cells that are adherent to the capillary wall. The latter is diagnostic of lymphovascular invasion and is the most reliable criterion for malignancy (carcinoma) despite the absence of necrosis, cytological atypia, and mitotic activity.

Considering the pathological findings, a PET scan was performed on 25/07/2022. The scan showed postsurgical changes, including surgical clips and linear FDG uptake at the operative bed, likely attributable to postoperative inflammation. No evidence of FDG–avid regional or distant disease was detected. Genetic testing for *CDC73* gene mutations was not performed due to lack of insurance coverage and financial constraints.

Follow‐up blood test results are shown below.

**Table:** 

Date	PTH (15–65 pg/mL)	Calcium (8.6–10.4 mg/dL)	Phosphorus (2.7–4.5 mg/dL)	Creatinine (0.4–1 mg/dL)
20/07/2022	105	9.4	2.8	—
15/11/2022	109	9.4	2.8	1.88
20/02/2023	120.6	9.4	2.5	1.76
03/11/2023	81.9	9.6	2.6	1.7

## 3. Discussion

Parathyroid carcinoma is a rare cause of PHPT (<1%) though some reports cite incidences up to 5.2% [8]. Unlike parathyroid adenomas, which predominantly affect women, PC shows no gender predilection and is typically diagnosed a decade earlier, around the mid‐40 s compared to the mid‐50 s for adenomas [9].

The etiology of PC involves a combination of genetic and environmental factors. Childhood exposure to head and neck radiation and end‐stage renal disease are potential risk factors, though their exact roles remain unclear [10]. Although most of the PC cases occur sporadically, the disease may be associated with syndromic entities, namely, HPT‐JT syndrome, MEN1, MEN2A, and others.

Advancements in genetic research have enhanced our understanding of PC pathogenesis, particularly in the context of HPT‐JT syndrome, where patients develop PHPT from both benign and malignant parathyroid tumors, as well as other neoplasms such as those affecting the maxilla and uterus [11, 12]. Pathogenic variants in the *CDC73* tumor suppressor gene (formerly HRPT2), located on chromosome 1, play a central role in PC pathogenesis. *CDC73* encodes parafibromin, a nuclear protein involved in chromosome remodeling, gene expression regulation, and inhibition of cell proliferation [13]. Germline inactivating mutations in *CDC73* with subsequent loss of parafibromin expression are present in over 50% of HPT‐JT families. The discrepancy between the frequency of PC in HPT‐JT (up to 15%) and PHPT (<1%) is partly explained by *CDC73* mutations found in up to 80% of sporadic PC cases [14]. Conversely, other germline mutations predisposing to parathyroid adenomas, such as MEN1, CASR, and FHH, are rarely implicated in PC [15]. Additional genetic alterations seen in sporadic PC include mutations in the retinoblastoma (*Rb*), *p53*, breast carcinoma susceptibility (*BRCA2*), and cyclin D1/parathyroid adenomatosis gene 1 (*PRAD 1*) genes [16].

While symptoms overlap between benign and malignant parathyroid disease, certain clinical and biochemical features may increase suspicion for PC. The disease typically follows an indolent course with low malignant potential but causes significant morbidity due to hypercalcemia‐related complications rather than tumor spread [17]. Since PCs are mostly functioning, patients typically present with more symptomatic hypercalcemia compared to those with parathyroid adenomas, with serum calcium levels often exceeding 14 mg/dL and serum PTH concentrations usually 5–10 times the upper limit of normal. These patients may exhibit various symptoms such as gastrointestinal complaints (nausea, vomiting, abdominal pain, and constipation), polyuria, polydipsia, fatigue, myopathy, disorientation, and neurocognitive deficits. Additionally, patients with PC are more likely to exhibit significant bone and kidney involvement such as renal failure, pathologic fractures, nephrolithiasis, and occasionally osteitis fibrosa cystica [9, 18]. Bone involvement can be reflected by a markedly elevated serum total alkaline phosphatase in the absence of liver disease. Moreover, PC can present with a parathyroid crisis or life‐threatening hypercalcemia, leading to renal failure, cardiac arrhythmia, and neurological manifestations such as stupor, altered sensorium, or coma [9]. The exact mechanism linking hypercalcemia to psychosis remains unclear but may involve disruptions in central neurotransmitters like dopamine and serotonin, as well as glutamate‐mediated excitotoxicity. These disturbances, particularly in cases related to parathyroid adenoma, appear to resolve after surgical treatment, suggesting a reversible neurochemical basis for the psychiatric symptoms [19, 20]. Other potential findings include pancreatitis, peptic ulcers, and anemia. Unilateral vocal cord palsy and/or palpable neck mass in hypercalcemic patients may raise the suspicion of PC [9]. Rarely, PCs are nonfunctioning [21]. In such cases, patients may remain normocalcemic and present with a neck mass, often at a more advanced stage of disease. These tumors tend to be more aggressive, affecting surrounding structures such as the recurrent laryngeal nerve and esophagus, and are more prone to regional and distant metastasis [22].

In addition to the previously mentioned biochemical findings, such as high calcium and PTH levels, markedly elevated total alkaline phosphatase, and reduced glomerular filtration rate, serum and urinary human chorionic gonadotropin (hCG) levels have been reported to be elevated in PC but not in patients with parathyroid adenoma [23]. hCG, particularly its hyperglycosylated isoform which has been recognized in both trophoblastic and certain nontrophoblastic cancers, has shown potential as a biomarker for distinguishing parathyroid carcinoma from benign PHPT, with elevated levels more frequently observed in malignant cases. However, due to limited and inconsistent data, further large‐scale studies are needed to validate its diagnostic and prognostic value [24].

Imaging studies are performed before surgery to locate the disease and determine its extension. These studies include parathyroid ultrasound, Tc99m sestamibi scintigraphy, computed tomography (CT), magnetic resonance imaging (MRI), and positron emission tomography (PET). Ultrasound findings that raise suspicion of PC include a large (greater than 3cm), lobulated, hypoechoic, or heterogeneous parathyroid gland with poorly defined borders, a thick capsule, suspicious vascularity, calcifications, and sometimes cervical lymph node involvement [25]. Scintigraphy cannot reliably distinguish benign from malignant lesions. CT and MRI are useful in localizing the tumoral lesion as well as identifying the invasion of surrounding structures and detecting distant metastasis. PET/CT 18‐FDG is used to evaluate for the extension of PC and its recurrence; however, it has low sensitivity in identifying small lesions [25–27]. If a parathyroid lesion is suspected to be malignant, fine needle aspiration is discouraged due to the risk of tumor seeding along the biopsy tract and the inability to definitively distinguish PC from benign lesions [28].

Despite suggestive clinical and imaging findings, a definitive diagnosis of PC is made histologically. Schantz and Castleman [6] in 1973 defined the histological criteria for PC and included sheets or lobules of tumor cells with interspersed fibrous bands, mitotic figures, necrosis, and capsular and vascular invasion. Of these, capsular and vascular invasion are the most reliable indicators of malignancy [29]. Tumors exhibiting atypical histologic features without clear evidence of capsular, vascular, and/or perineural invasion are classified as atypical adenomas [30].

Because available data on tumor characteristics and prognosis are still limited, a formal staging system does not currently exist. However, the newest release of the Tumor, Node, and Metastases (TNM) cancer manual from the combined American Joint Committee on Cancer (AJCC) and the Union for International Cancer Control (UICC) has proposed collecting data on variables such as patient demographics, genetic mutation, tumor size, invasion status, and histologic features to facilitate future staging efforts [31, 32]. Genetic testing for germline CDC73 pathogenic variants is appropriate in most patients with sporadic parathyroid carcinoma, particularly when there are family members who may benefit from genetic counseling. It is also indicated when the carcinoma occurs in the context of known familial hyperparathyroidism or when clinical features suggestive of HPT‐JT syndrome are present in the index patient [33].

Surgical resection remains the only curative treatment for PC. Preoperative goals include anatomical localization and correction of hypercalcemia. The standard approach is en bloc excision, involving removal of the affected parathyroid gland, ipsilateral thyroid lobe, and any involved tissues or lymph nodes. Avoiding capsular rupture is crucial to prevent recurrence [34]. According to the national cancer database survey, the 10‐year survival for patients with an incomplete resection was 49%, while those with a complete resection was 66% [35]. Postoperative hypocalcemia may indicate successful tumor removal.

While some consider radiotherapy effective as a complementary treatment, there is consensus that chemotherapy is ineffective. It is well known that PCs are generally not radiosensitive, except when used postoperatively to prevent regrowth. A study conducted by Busaidy et al. followed 27 patients for 7.9 years and found that among those who received adjuvant radiotherapy, only one out of six experienced local recurrences. In contrast, 10 out of 20 patients who did not receive adjuvant radiotherapy had local recurrence [36].

In inoperable cases, prognosis is poor, but hypercalcemia can be managed medically using agents that inhibit osteoclastic bone resorption. Bisphosphonates are the treatment of choice for recurrent and metastatic disease, with zoledronic acid normalizing calcium in 80%–100% of patients. Calcitonin is another option for early treatment to lower calcium levels until other hypocalcemic agents take effect. Denosumab can also be used as an alternative therapy. Calcimimetics have shown efficacy in two‐thirds of inoperable PC cases. For patients who are resistant to hypocalcemic agents, immunization with human and bovine PTH peptides presents a novel and promising approach [34].

**Figure 3:**
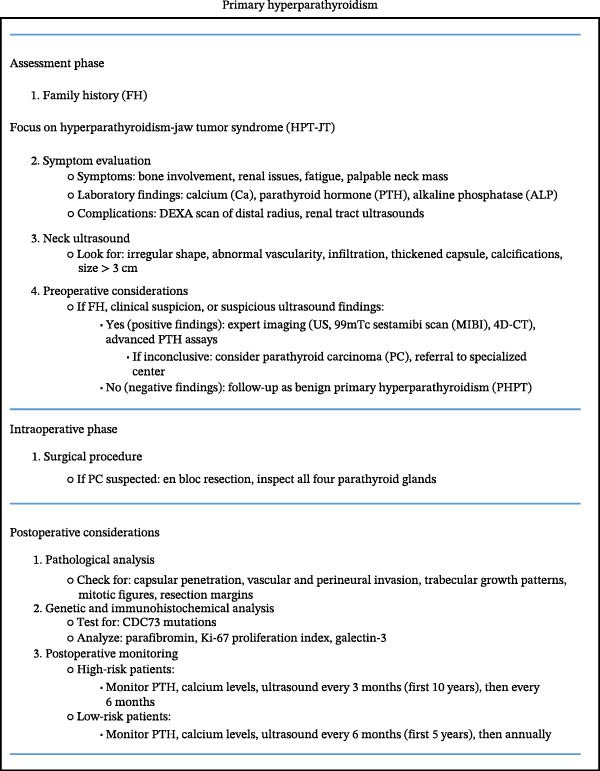
Suggested approach to patients with parathyroid carcinoma.

PC typically follows an indolent but progressive course. While some cases relapse within 2–3 years after surgery, recurrences have also been reported as late as 23 years postoperatively. Lifelong monitoring is essential, with calcium and PTH levels measured biannually for the first 5 years and then annually thereafter. Additionally, a neck ultrasound is performed annually [31, 37]. Roser et al. [38] proposed a diagnostic algorithm for PHPT suggesting that PC should be considered if there is a history of HPT‐JT syndrome or high calcium and PTH levels. The algorithm is outlined in Figure 3 [38].

Novel targeted therapies based on molecular pathogenesis studies represent a promising future for PC management. Molecular alterations in patients with advanced PC can be identified to guide treatment. In a study by Teleanu et al. that employed whole‐genome and RNA sequencing, major findings included the identification of *APOBEC* (apolipoprotein B mRNA editing enzyme and catalytic polypeptide‐like) overactivation and recurrent overexpression of *RET* (Ret Proto‐Oncogene) and *FGFR1* (Fibroblast Growth Factor Receptor 1). Targeted therapy in two patients with an immune checkpoint inhibitor, tyrosine kinase inhibitor, and PARP (Poly(ADP‐Ribose) Polymerase) inhibitor showed a biochemical response and progression‐free disease, thus demonstrating the potential benefits of comprehensive molecular profiling in PC management [39].

In the presented case, there was a high index of suspicion for parathyroid carcinoma given the severity of the clinical presentation, which included hypercalcemic crisis with psychosis and muscle weakness, alongside markedly elevated calcium and PTH levels. Initial management with aggressive intravenous fluids resulted in partial improvement of calcium levels, though they remained mildly elevated. Due to the patient’s renal impairment (creatinine clearance of 21 mL/min), intravenous bisphosphonates were contraindicated. Additionally, calcitonin was unavailable at the time. As a result, denosumab, a RANKL receptor inhibitor typically used for osteoporosis and malignancy‐associated hypercalcemia [40], was selected as an alternative. Its use in refractory hypercalcemia secondary to parathyroid carcinoma has been reported in several case studies [41]. Although the standard dose ranges from 60 to 120 mg subcutaneously, a reduced dose of 30 mg was administered following multidisciplinary discussion between endocrinology and nephrology teams to minimize the risk of hypocalcemia, a known adverse effect, particularly in patients with renal dysfunction [42]. Intraoperatively, the only notable finding was an enlarged right inferior parathyroid gland. Local excision was performed based on the frozen section result and an appropriate intraoperative PTH drop. Although frozen section analysis suggested a parathyroid adenoma, final histopathology confirmed a diagnosis of parathyroid carcinoma with low cytonuclear grade. Differentiating parathyroid carcinoma from benign lesions is well recognized as a diagnostic challenge [43].

While en bloc resection remains the gold standard surgical approach as it ensures complete tumor removal with clear margins and reduces the risk of recurrence, the optimal surgical strategy for parathyroid carcinoma is still debated. Some studies advocate for en bloc resection due to improved outcomes including lower recurrence rates and longer disease‐free survival, whereas others propose that a more conservative excision may be appropriate in selected cases [37]. In this case, given the postoperative drop in PTH levels, maintained normocalcemia, negative surgical margins, and a negative PET scan, no further surgical intervention was undertaken. The patient was instead managed with active surveillance. On follow‐up, PTH levels remained mildly elevated, but calcium levels were stable within the normal range, and the patient remained asymptomatic. Genetic testing would have been ideal, particularly given the family history of nephrolithiasis; however, it was not performed due to lack of insurance coverage and financial limitations.

## 4. Conclusion

PC, though a rare cause of hyperparathyroidism, poses significant diagnostic challenges due to its often indistinguishable clinical and biochemical presentation from benign disease. Early identification of at‐risk patients is essential, as surgery remains the primary and most effective treatment, particularly given the potentially fatal complications of uncontrolled hypercalcemia. In cases of hypercalcemic crisis, surgery should be delayed until metabolic abnormalities are medically corrected. Long‐term follow‐up is critical, and while curative treatment is often surgical, palliative options for inoperable cases are limited. Genetic testing, particularly for CDC73 mutations, may support early detection and monitoring, and emerging therapies such as immunotherapy offer hope for future advances in management. Lessons learned from this case highlight the difficulty in distinguishing parathyroid carcinoma from benign disease and the continued debate surrounding the optimal surgical approach, even though en bloc resection is generally associated with improved outcomes. This case also demonstrates that active surveillance can be an appropriate strategy when en bloc resection is not performed but postoperative findings such as negative margins, stable normocalcemia, and nonprogressive PTH levels are reassuring.

## Author Contributions

Helena Fahmi and Mahmoud Karaki contributed equally to the clinical evaluation, management, and drafting of the case report, serving as cofirst authors. Paola Atallah, as the corresponding author, supervised the preparation of the manuscript and coordinated the submission process. Layal Akl and Fatima Yasmin assisted with data collection, literature review, and editing of the manuscript.

## Funding

No funding was received for this manuscript.

## Disclosure

All authors reviewed and approved the final version of the case report.

## Consent

Written informed consent was obtained from the patient for publication of this case report and any accompanying images.

## Conflicts of Interest

The authors declare no conflicts of interest.

## Data Availability

The data that support the findings of this study are available in the supporting information of this article.
